# Perceived user preferences and usability evaluation of mainstream wearable devices for health monitoring

**DOI:** 10.7717/peerj.5350

**Published:** 2018-07-25

**Authors:** Yuxi Jia, Wei Wang, Dong Wen, Lizhong Liang, Li Gao, Jianbo Lei

**Affiliations:** 1 Department of Medical Informatics, School of Public Health, Jilin University, Changchun, Jilin Province, China; 2 Peking University Third Hospital, Beijing, China; 3 The Affiliated Hospital of Guangdong Medical University, Zhanjiang, Guangdong Province, China; 4 Peking University School of Stomatology, Beijing, China; 5 Center for Medical Informatics, Peking University, Beijing, China; 6 School of Medical Informatics and Engineering, Southwest Medical University, Luzhou, Sichuan Province, China

**Keywords:** Usability evaluation, User experience, Fitness tracker, Wearable device

## Abstract

**Background:**

There are many problems with fitness trackers, such as device usability, which limit their large-scale application, and relevant studies are limited in terms of their sample size and evaluation methods. The purpose of the study was to evaluate the perceived usability of various mainstream fitness trackers on the market, and to learn about user feedback on feature preferences for each device.

**Methods:**

Trial use of seven mainstream fitness trackers (two smart watches and five smart wristbands) followed by a survey study were applied. The questionnaire was specifically developed for this study, which included two parts (user preferences and device usability in five dimensions). We recruited users to test the devices for at least 30 days and asked experienced users to provide feedback in order to evaluate each device, including the rating and user preference of each device.

**Results:**

We received 388 valid questionnaires, in which users rated their responses on a five-point Likert scale. (1) User preference: the average user satisfaction was 3.50–3.86 (points), and the rating for willingness to buy averaged between 3.36 and 3.59. More users were willing to wear (58.3–81.3%) and purchase (56.8–83.0%) the devices than were not. The top three general feature preferences were daily activity tracking, heart health monitoring, and professional fitness tracking. The top three health-related feature preferences were heart rate monitoring, daily pedometer, and professional fitness tracking. (2) Usability evaluation: product design was rated from 3.57 to 4.00; durability, 3.63–4.26; ease of use, 3.70–3.90; added features, 3.30–3.83; and user-rated accuracy, 3.44–3.78. A significant difference was observed in the rating of product design and durability among the different devices (*p* < 0.05) score.

**Conclusions:**

Users generally had positive subjective intent regarding fitness trackers but were less satisfied with their cost effectiveness. The users preferred health related features such as heart health monitoring, and professional fitness tracking. The rating of most of the current mainstream fitness trackers was fair with some significant differences among the devices. Thus, further improvement is needed.

## Introduction

Wearable devices, which are equipped with innovative features, represent one of the latest directions in hardware development. A wearable device refers to a wearable micro-electronic device that does not affect the daily activities of the user while monitoring specific quantities of interest (e.g., air quality, radiation exposure, heart rate) and/or providing therapeutics (heart pacing, insulin infusion). Through embedded electronics and software applications, such as computer chips, sensors, and liquid crystal display screens, wearable devices enable innovative features such as user condition collection, real-time information support, and cloud interaction (Wikipedia, 2018). In the 1980s, Steve Mann invented the first wearable computer, a backpack helmet computer, and is thus known as the “father of wearable computers.” During the following decades, wearable computers, such as wearable wireless cameras and digital pacemakers, were successively introduced. The development of wearable devices was closely related to pervasive computing, wearable computers, and networking technologies. Pervasive computing allows information gathering and processing at any time, at any place, and in any manner. Networking refers to the network connection through which embedded electronics, sensors, software, and connected physical devices and cars that are connected to the network to allow for data collection and exchange in the network (Wikipedia, 2017). Wearable devices result from technological advances in hardware performance and computational theory, with innovative user interactions, computational speeds, and content support. Currently, common wearable devices include smart wristbands (such as the Fitbit), smart watches (such as the Apple Watch), smart glasses (such as Google Glass), and virtual reality/augmented reality (AR) devices (such as Microsoft HoloLens).

Wearable devices have attracted much attention in the market and have many potential applications, particularly in healthcare. The market for wearable devices is growing quickly. In 2017, the vendors of worldwide wearable device tracker shipped a total of 125.5 million wearable devices, marking a 20.4% increase from the 104.3 million units shipped in 2016. From there, the wearables market will nearly double before reaching a total of 240.1 million units shipped in 2021, resulting in a 5-year compound annual growth rate of 18.2% (IDC, 2017b). China is one of the largest markets for wearable devices, and the market revenue is projected to reach 39.4 billion Yuan ($5.74 billion or €5.41 billion) in 2018 (Analysys, 2017; CCSInsight, 2016; Gartner, 2016). In the fourth quarter of 2017, 37.9 million wearable devices were shipped worldwide, which was 8% higher than in the same period of the previous year. The top three brands were Apple, Fitbit, Xiaomi with 21.0%, 14.2%, and 13.0% of the market share (IDC, 2017a). The application prospects for innovative wearable devices are driving their remarkable market performance. Wearable devices use sensors to collect personal data and data from the surrounding environment, Bluetooth or a 4G network to exchange information, and a touch screen, eye tracking, gestures, and speech recognition to interact (Xie & Zhang, 2015). As a result, wearable devices have greatly improved user experiences and information exchange capabilities relative to conventional electronic devices. Thanks to these characteristics, wearable devices have broader prospects in functional applications, such as real-time warnings of health risks and AR experiences. In terms of interaction, users are more naturally integrated with wearable devices, such as real-time information retrieval through smart glasses. The content of information exchange with wearable devices is more comprehensive, and a network of many wearable devices enables complex characterization of the individuals and the environment, which enables a more personalized and specific service experience (Wu et al., 2016). Wearable devices have broad prospects in healthcare applications such as real-time vital sign and health status monitoring of elderly patients with Parkinson’s, heart diseases, or other chronic diseases (Lu et al., 2016) and can collect important and trivial data about patient position and lifestyle more easily and accurately. In addition, interaction through wearable devices can be integrated with life scenarios to provide better health education (Sultan, 2015).

Although the application prospects are broadening for wearable devices, some people still have concerns about these devices, particularly their usability. Features about wearable devices other than usability were extensively studied such as reliability and accuracy evaluation of wearable devices. The accuracy of fitness tracking and activity monitoring results still plays a major role in health promotion: Xie et al. found mainstream devices are able to reliably measure heart rate, number of steps, distance, and sleep duration, but the measurement accuracy of energy consumption is still inadequate. Dondzila et al. examined two wrist-worn activity trackers, the Fitbit and Mio Fuse detected steps and predicted energy expenditure (EE) with heightened precision as exercise intensity increased; Rozanski et al. determined the accuracy of two wrist-worn fitness trackers for stroke patients; in their reliability study, Wen et al. found the wearable devices are only reliable in measuring the number of steps and distance, which can be used as health assessment indicators (Dondzila et al., 2018; Rozanski et al., 2018; Wen et al., 2017; Xie et al., 2018). However, EE, sleep quality, and so on, are still inadequate. In addition, the effectiveness and safety of wearable devices must be further improved. For example, more wearable devices may result in busy communications, the security of personal information is an important issue in the network environment, and data hacking may harm the user’s physical and mental health (Austen, 2015; Mills et al., 2016; Tranter, 2018). In addition to problems related to these functional features, few studies have been conducted to investigate the usability of wearable devices. Lu et al. showed that all available application studies on clinical health monitoring did not test device usability before the start of the interventions, which may affect intervention outcomes and risk control. Usability tests mainly include research level device comparisons and market-oriented product evaluations. Schenkenfelder et al. tested 18 subjects on three representative products (smart glasses, smart watches, smart wristbands) for jogging. After jogging, the System Usability Scale (SUS) was used to rate usability, and facial expression cards and a pleasure, arousal, and dominance emotional scale were used to determine user experience. The results showed that Google Glass was rated low on all items (mean SUS score: 51). The LG G watch and Runtastic Orbit showed good and comparable usability. The user experience was more positive with smart watches and smart wristbands than with smart glasses. Kaewkannate et al. rated seven users’ experiences with four smart wristbands after testing each smart wristband for 1 week. The authors used a home-designed questionnaire to rate user satisfaction on five aspects (design, battery, data synchronization, interface, and ease of use) and four features (pedometer, sleep monitoring, nutritional analysis, and distance monitoring). The results showed that the users were most satisfied with Withings Pulse and least satisfied with Fitbit Flex. For product features, the users were most satisfied with the pedometer and least satisfied with the nutritional analysis (Kaewkannate & Kim, 2016; Lu et al., 2016; Schenkenfelder & Selinger, 2016). In addition, more diverse and timely evaluations of wearable devices are mainly from product reviews on tech news websites such as CNET and Engadget. These product reviews involve user experience and personal opinions, which is the basis for the official rating from the website. These product reviews provide a reference for user experience from additional perspectives, but they mainly reflect subjective feelings, contain no quantitative data, and lack unified rating criteria.

Wearable devices used for activity and vital sign monitoring are generally called fitness trackers. This study took fitness trackers as the main research objects. Given the lack of unified rating criteria and small sample sizes in current studies on usability evaluation and applications of fitness trackers, in this study, we selected representative products from two types of fitness trackers (smart watches and smart wristbands) and included well-known international and Chinese smart wristbands by taking into account the market situation in China. We analyzed the features and functions of each fitness tracker and designed a questionnaire that comprised two parts: product features and user preferences. We recruited more subjects than any previous study (Kaewkannate & Kim, 2016; Ridgers et al., 2018; Ryu & Smith-Jackson, 2006) to rate the user experience of each mainstream fitness tracker. The aim of the study was to explore user evaluation methods of fitness trackers, evaluate current popular fitness trackers on the market, and analyze existing usability problems with fitness trackers.

Traditional research methods for product features and user preferences often use thematic analysis approaches and then summarization of the results (Mercer et al., 2016; Nguyen et al., 2017; Papi, Murtagh & McGregor, 2016). Although such methods are popular, they are still having some limitations such as the single type of survey results and no effective data interpretation, etc. Results obtained by this method are difficult to interpret or translate into practice (Ho, 2017). The most common strategy for constructing this type of evaluation is the use of quantitative surveys, particularly Likert-type scales. Likert-type scales are one of the most commonly used psychometric scales for examining self-reported perceptions and attitudes where respondents complete a list of survey items that are summed or averaged to produce a numerical score. Therefore, we attempted to apply a five-point Likert scale to evaluate user preferences. At the same time, the results of content and user preferences are visualized to avoid the option interference caused by the questionnaire approach (Hartley & Betts, 2010).

## Methods

### Device selection and subject recruitment

Representative devices were selected following a market analysis. We referenced the market ranking data in the market research reports of fitness trackers released in the past 2 years by Canalys, the shipment of fitness trackers was led by Fitbit, Jawbone, and Mi. In addition, the shipment of smartphone which is closely related to wearable device was led by Apple, Samsung, and HUAWEI (Canalys, 2014, 2015). Therefore we selected and included current mainstream market-leading fitness trackers, including Samsung Gear S (Samsung Electronics Co., Ltd, Suwon, South Korea), Apple Watch (Apple Inc., Cupertino, CA, USA), Fitbit Surge (Fitbit Inc., San Francisco, CA, USA), Jawbone Up3 (Jawbone Inc., San Francisco, CA, USA), Mi Band (Xiaomi Inc., Beijing, China), HUAWEI Talk Band B2 (Huawei Technologies Co., Ltd, Shenzhen, Guangdong, China), and Misfit Shine (Misfit, Inc., Burlingame, CA, USA) in this usability evaluation study. Of the selected devices, Apple Watch and Samsung Gear S were representative products from leading manufacturers of mobile digital devices; Fitbit Surge, Jawbone Up3, and Misfit Shine were well-known international exercise wristbands at different price levels; and Mi Band and HUAWEI TalkBand B2 were leading exercise wristbands on the Chinese market.

The target population of this study was people who were interested in wearable devices and desired to improve health status with fitness trackers. We recruited two groups of subjects: trial users and experienced users. On one hand, we purchased three units of each type of device and recruited trial users from colleagues and the community through social networks and public posters. In order to ensure a thorough and responsible experience of the test devices, the subject chose a device they are interested in themselves. After instructing the users on how to use the device, we asked the users to continuously use the device for 30 days intensively in order to become familiar with its features. At the end of the trial, the subjects were asked to complete an evaluation questionnaire to rate the trial products. On the other hand, using snowball sampling, we sampled experienced users who used devices we studied at least longer than 1 month from social networks such as our WeChat circle of friends and asked them to complete an evaluation questionnaire in order to collect data. This study started from October 2015 to recruit users to trial, and finished by October 2016. This research has passed an audit of the Peking University Biomedical Ethics Committee No. IRB00001052-16008-Exampt. We obtained written informed consent from all participants.

### Questionnaire design

We did not find any established scale for evaluating fitness trackers; therefore, in order to achieve better validity of the questionnaire, we referenced the following resources. First, the usability of a device can be examined using recognized dimensions in the literature on the features of fitness trackers, such as the five features of wearable computers (mobility; wearability; constancy; hands-free or one-hand operation; and AR, mediation, and context awareness) described in Bass (1997). Second, several classical usability scales have been used to evaluate software systems, such as the After Scenario Questionnaire (Lewis, 1992), the Computer System Usability Questionnaire (Baily, 1989), the Post-study System Measurement Inventory (Lewis, 1992), the Software Usability Measurement Inventory (Kirakowski & Corbett, 1993), and the SUS (Brooke, 1996). Third, the Mobile Phone Usability Questionnaire (Ryu & Smith-Jackson, 2006) is an established scale for evaluating hardware and has been translated into Chinese; however, most of the questions are related to phone features and thus cannot reflect the features of fitness trackers. On the basis of the above references, we designed our own feedback questionnaire to evaluate fitness trackers. The contents of the questionnaire were further revised and validated through expert review and pilot study. We included questions about the user’s preferences in terms of the desired user experiences, product features and the use and purchase of each device. For device usability we designed a five-point Likert scale, which assessed both the hardware and software experience in 20 questions on five dimensions (product design, durability, ease of use, added features, and user-rated accuracy).

### Data collection and analysis

Questionnaires were answered online and the results were collected through the website Sojump, one of the world’s largest free survey platform. The users could access and complete the questionnaire, “smart fitness tracker comprehensive evaluation questionnaire” (http://www.sojump.com/jq/5733344.aspx), on a PC or mobile device, and then, the data were analyzed. To ensure data safety, the data were stored locally with a cloud backup. For multiple choice questions, the percentage of users who chose each device was statistically analyzed to determine the level of user acceptance. The composite score of each fitness tracker was plotted on a radar graph to show the score distribution, and an analysis of variance was performed to analyze the mean score of each dimension of the different fitness trackers. For the feature preference questions, the mean weighted score ((Σ frequency × weighted value)/the number of responses) was calculated to rank the user preference for each feature.

## Results

### Demographic information of the subjects

In this study, we received a total of 388 completed questionnaires and 388 questionnaires were valid, the response rate was 100%. This is because the methods of recruiting respondents are mainly to roll snowball through the personal friends of researchers’ social media, and to recruit the volunteers who will try the devices before they agree to complete the questionnaire, the two methods are more likely to allow the subjects to complete the questionnaire according to prior agreement. The basic information of the subjects is shown in Table 1. The subjects are mainly from China, distributed in 28 provinces of China. In addition, there are 10 responses from USA, Singapore, Italy, and Canada. The subjects included more men (66.2%) than women. Most of the subjects were young, with the highest percentage of subjects in the 31–40 age group (29.1%), followed by the 18–25 age group (27.6%) and the 26–30 age group (22.7%). The majority of the subjects had completed an undergraduate education (47.7%), followed by postgraduate (master’s degree) education (25.3%). The majority of the subjects worked in the information technology (IT) Internet (29.4%) and healthcare (23.7%) industries. Furthermore, the largest percentage of the subjects earned ≥7,000 Yuan per month (48.7%), followed by 4,000–7,000 Yuan (24.0%).

**Table 1:** Basic information of the subjects.

	Frequency	Percentage (%)
**Device**		
Apple Watch	83	21.4
Samsung Gear S	36	9.3
Fitbit Surge	37	9.5
Jawbone Up3	32	8.2
Mi Band	122	31.4
HUAWEI TalkBand B2	47	12.1
Misfit Shine	31	8.0
**Gender**		
Male	257	66.2
Female	131	33.8
**Age**		
<18	10	2.6
18–25	107	27.6
26–30	88	22.7
31–40	113	29.1
41–50	56	14.4
51–60	13	3.4
>60	1	0.3
**Education level**		
Junior high school and below	3	0.8
Secondary school and senior high school	19	4.9
College (associate degree)	46	11.9
Undergraduate	185	47.7
Postgraduate (master’s degree)	98	25.3
Postgraduate (doctoral degree)	37	9.5
**Industry**		
IT internet	114	29.4
Finance	28	7.2
Healthcare	92	23.7
Education	29	7.5
Service	20	5.2
Manufacturing	19	4.9
Other	86	22.1
**Monthly income (Yuan)**		
<1,000	27	7.0
1,000–2,000	34	8.8
2,001–4,000	45	11.6
4,001–7,000	93	24.0
>7,000	189	48.7

### User needs and feature preferences

**Figure 1:**
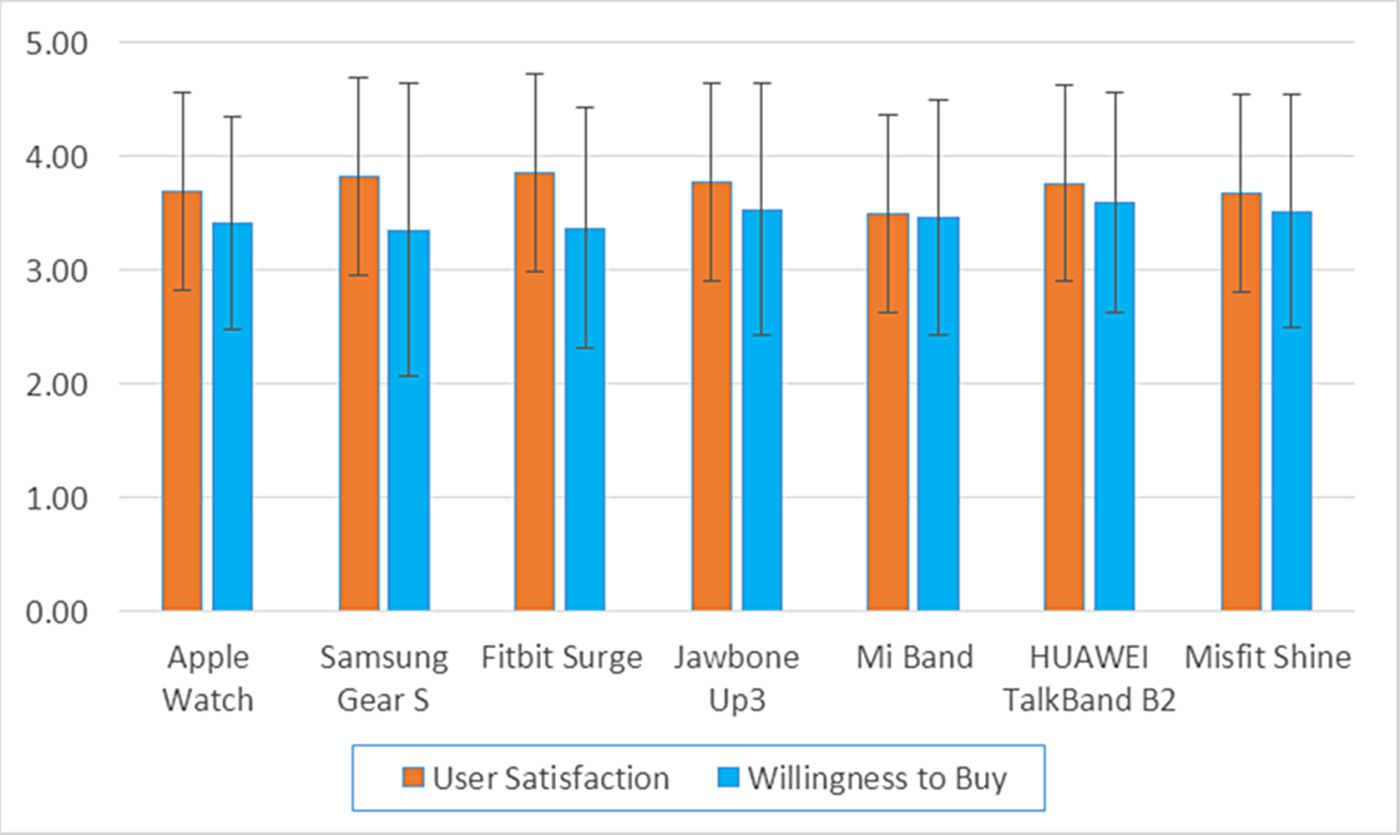
The mean satisfaction and willingness to buy scores for each device.

A five-point Likert scale (1: strongly dissatisfied, 2: dissatisfied, 3: neutral, 4: satisfied, 5: strongly satisfied) was used to rate the user’s satisfaction and willingness to buy each device, and the mean score of each device is shown in Fig. 1. The results showed that the average satisfaction score for the various devices ranged from 3.5 to 4, indicating near satisfaction with and no significant difference among the different devices. The satisfaction score was the highest for the Fitbit Surge (3.86 ± 0.95) and the lowest for the Mi Band (3.50 ± 0.86). The mean score of willingness to buy (three to four) was lower than the satisfaction score for all devices, which indicated that for each device, the users were either willing to buy or generally willing to buy with no significant difference among the different devices. The willingness to buy score was the highest for the HUAWEI TalkBand B2 (3.59 ± 0.97) and lowest for the Samsung Gear S (3.36 ± 1.29).

User willingness to wear and buy each device is shown in Table 2. For all devices, more users were willing to wear and buy the devices than not. For willingness to wear, the highest percentage of users were willing to wear the Jawbone Up3 (81.3%), and the lowest percentage were willing to wear the Samsung Gear S (58.3%). For willingness to buy, the highest percentage of users were willing to buy the HUAWEI TalkBand B2 (83.0%), and the lowest percentage were willing to buy the Fitbit Surge (56.8%). The Mi bracelet and HUAWEI TalkBand B2 were the only devices for which the percentage of users that were willing to buy the device was higher than those willing to wear the device.

**Table 2:** The percentage of subjects who were willing to wear and buy each device (%).

		Apple Watch	Samsung Gear S	Fitbit Surge	Jawbone Up3	Mi Band	HUAWEI TalkBand B2	Misfit Shine
Willingness to wear	Willing	74.7	58.3	70.3	81.3	77.9	74.5	80.6
	Unwilling	25.3	41.7	29.7	18.8	22.1	25.5	19.4
Willingness to buy	Willing	63.9	58.3	56.8	71.9	82.8	83.0	74.2
	Unwilling	36.1	41.7	43.2	28.1	17.2	17.0	25.8

The user preferences for the all possible features exist in current devices of each device are shown in Table 3, and the user preferences for the healthcare monitoring related features are shown in Table 4. Among the features, which included smart phone aid, entertainment, activity monitoring, and health monitoring, the top three preferences of users were daily activity tracking, heart health monitoring, and professional fitness tracking. For the healthcare features, the top three preferences of users were heart rate monitoring, daily pedometer, and professional fitness tracking.

**Table 3:** Preferences of users for device features.

Item	Number of user selections (weighted)
Daily activity tracking	7.33
Heart health monitoring	7.22
Professional fitness tracking	7.02
Sleep monitoring	7.01
Other vital signs monitoring	6.99
Diet tracking	6.44
Data trend statistics	6.29
Smart fitness coaching	5.67
Healthy lifestyle reminders	5.42
Social sharing	4.88
Communication aid	4.86
Event arrangement	4.56
Entertainment	3.45
Smart voice assistant	3.10

**Table 4:** Preferences of users for health management features.

Item	Number of user selections (weighted)
Heart rate monitoring	4.73
Daily pedometer	4.45
Professional fitness tracking	4.18
Electrocardiography (ECG) monitoring	4.16
Blood oxygen saturation monitoring	3.91
Temperature monitoring	3.27
Blood glucose monitoring	2.91
Healthy lifestyle reminders	2.83

### Exploring usability evaluation methods for fitness trackers

**Figure 2:**
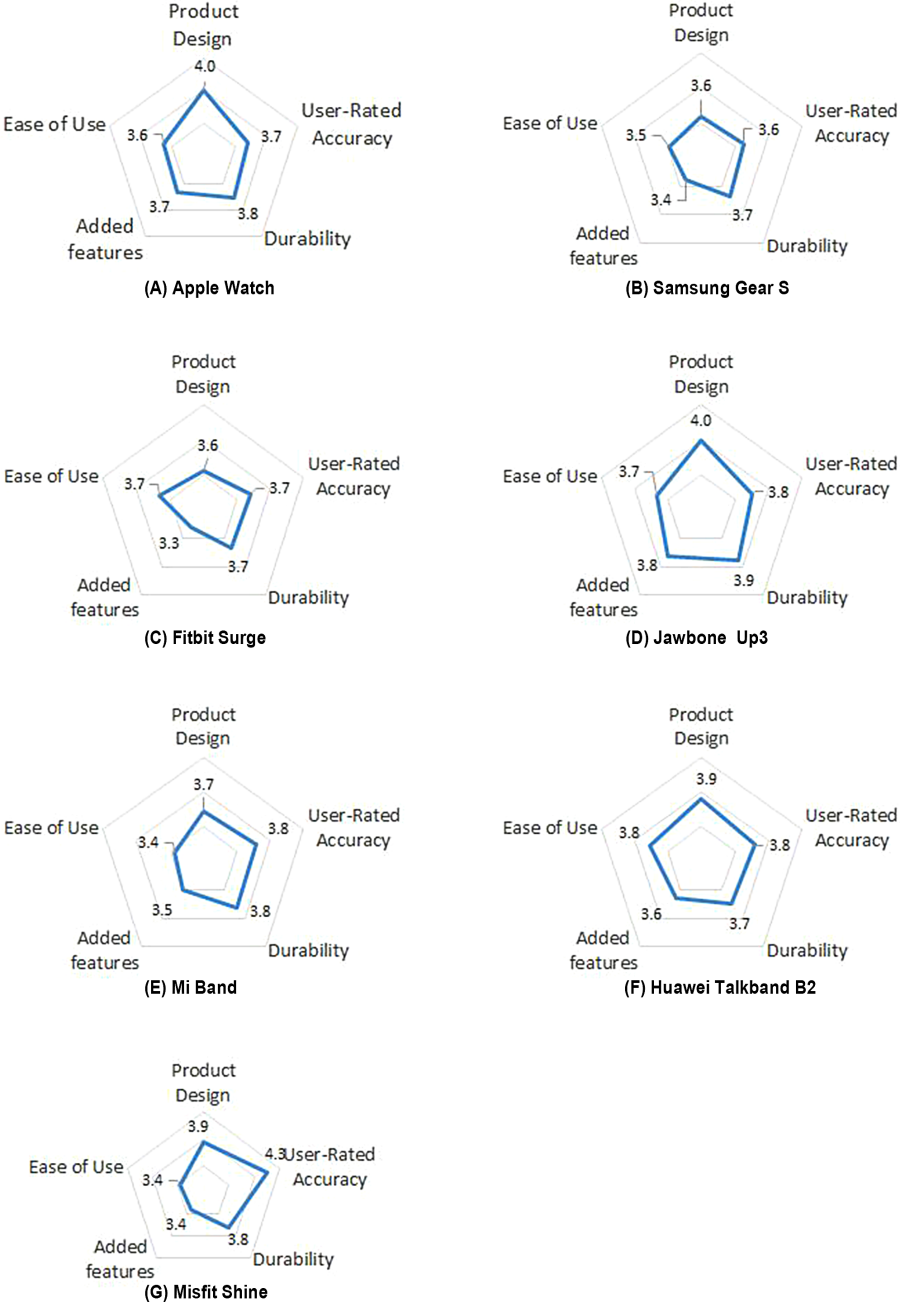
Radar graphs showing variations of five dimension scores of the 7 Devices.

Similarly, a five-point Likert scale (1: strongly dissatisfied, 2: dissatisfied, 3: neutral, 4: satisfied, 5: strongly satisfied) was used for the composite user ratings of each device. The score for each dimension for each device was plotted on a radar graph (Fig. 2). The results showed that the scores for each of the five dimensions varied among the different fitness trackers. Regarding product design, the highest score was for the (A) Apple Watch (4.00), and the lowest score was for the (C) Fitbit Surge (3.57). Regarding durability, the highest score was for the (G) Misfit Shine (4.26), and the lowest score was for the (B) Samsung Gear S (3.63). Regarding ease of use, the highest score was for the (D) Jawbone Up3 (3.90), and the lowest score was for the (C) Fitbit Surge (3.70). Regarding added features, the highest score was for the (D) Jawbone Up3 (3.83), and the lowest score was for the (C) Fitbit Surge (3.30). Finally, regarding user-rated accuracy, the highest score was for the (F) HUAWEI TalkBand B2 (3.78), and the lowest score was for the (E) Mi Band (3.44).

The analysis of variance of the scores for each device is shown in Table 5. A significant difference was observed in product design and durability among the different devices (*p* < 0.05). No significant differences were observed in ease of use, added features or user-rated accuracy among the different devices.

**Table 5:** Analysis of variance of the scores for each device.

	*F* (6, 382)	*p*-value
Product design	3.001	0.007
Durability	2.824	0.011
Ease of use	0.431	0.858
Added features	2.076	0.055
Accuracy	1.132	0.343

## Discussion

This study was unique in terms of the exploration of evaluation methods, the breadth of samples, and the diversity of the device selection but limited in terms of the instructions for completing the questionnaire and cross-validation of questionnaire validity. First, at present, no systematic, comprehensive, differential evaluation method with large-scale validation is available in scientific research or product reviews for evaluating the usability of fitness trackers. In this study, we conducted an extensive search of the literature and product reviews to design a questionnaire that included five dimensions (product design, durability, ease of use, added features, and user-rated accuracy) in order to comprehensively characterize each fitness tracker and visually demonstrate the pros and cons of each product using radar graphs. Second, to evaluate specific fitness trackers, in this study, we included the latest design of seven representative fitness trackers and sent out approximately 400 questionnaires. Moreover, given the continuous development of fitness trackers, some scholars also noted the importance of ongoing device evaluation (Schenkenfelder & Selinger, 2016). In this study, we selected the latest and most popular fitness trackers, including international and Chinese smart watches and smart wristbands, which covered a wide range and various types of devices. Taken together, this study is the first of its kind in terms of the number of subjects and the number and types of trial devices included. We compared various types of fitness trackers in order to identify the features of each device. Nevertheless, this study has certain limitations: first, no proven, effective, and specific usability evaluation scale is available for fitness trackers; thus, we referenced several similar evaluation scales and the literature on the features of fitness trackers to design our own questionnaire to evaluate fitness trackers. We included 20 items on five dimensions in the questionnaire; however, the instructions on how to complete certain items were unclear or did not provide specific examples, which may have hindered user comprehension of the items. Thus, in the future, we will further refine and validate the description of questionnaire items or use specific examples by conducting focus groups. Additionally, because of issues with user access to specific fitness trackers, in this study, we did not cross-validate the validity of different groups of questionnaires, which requires further research to develop more convincing usability evaluation benchmarks.

The main study findings comprised two parts. Users were generally unsatisfied with the cost-effectiveness of the current fitness trackers and rated most items as “fair.” Regarding user needs and preferences, this study showed that the user satisfaction and willingness to buy were between “unwilling” and “willing to buy” for all devices. Overall, the user satisfaction score was higher than the willingness to buy score, suggesting that users may be unsatisfied with the cost-effectiveness of the fitness trackers. Moreover, more users were willing to wear and buy the devices than not, and the two Chinese smart wristbands were the only devices for which the users were more willing to buy than wear the device, which was consistent with the cost-effectiveness results described above and demonstrated that the users were less willing to buy than to use the devices. For the device features, the users generally preferred daily activity and health monitoring, whereas for the healthcare features, the users generally preferred heart rate monitoring and fitness tracking. Furthermore, a significant difference was observed in the product design and durability rating between the different mainstream fitness trackers. These results suggest that the ratings of these three features varied among the different fitness trackers. No significant differences were observed in the ease of use or user-rated accuracy ratings, suggesting that these two features were comparable among the different fitness trackers. In this study, we were able to comprehensively and visually evaluate fitness trackers using a scale that included the five dimensions described above. Among all the usability evaluation dimensions, the lowest score was for added features of the Fitbit Surge (3.30), and the highest score was for durability of the Misfit Shine (4.26). In general, the score for each dimension was 4.0 or below for all the fitness trackers, suggesting that the devices failed to surprise users in certain aspects and that a lack of user stickiness resulted in less-than-ideal overall ratings, which should be heeded by manufacturers.

Evaluation results vary among other related evaluation methods, particularly among product reviews of fitness trackers from tech news websites, and we kept this in mind while developing our own questionnaire in this study. For fitness trackers on the market, the most diverse and timely product evaluations are product reviews from tech news websites such as CNET and Engadget. These news websites ask experienced electronic device users to use various products, introduce the features and user experience of various products to the audience, and give an official website rating. We gathered the official ratings of different devices from tech media websites (Table 6; updated: March 1, 2018), wherein the CNET, TechRadar, and ZOL ratings are on a five-point scale and the Engadget rating is on a 100-point scale that is converted to a five-point scale and the mean value then calculated. Table 6 shows that the ratings vary among the different media websites, which may be due to variabilities in the rating criteria and provides indirect evidence of why it is important to develop and establish a standard rating system. Moreover, the mean score was low for all the devices, with the exception of the Misfit Shine (mean score > 3), which was lower than the score observed in this study. These divergent results are probably related to different considerations between experienced users and general users. Given these results, our method can be further validated by giving different weights to experienced users versus general users to evaluate fitness trackers more rationally.

**Table 6:** Product reviews of fitness trackers from tech news media websites.

Device	CNET	Engadget	TechRadar	ZOL	Mean
Apple Watch	3.5	79	3.5	2.8	2.8
Samsung Gear S	3	65	3.5	3.8	2.7
Fitbit Surge	3	73	3.5	2.8	2.6
Jawbone Up3	3	78	3	4	2.8
Mi Band	3.5	–	–	3.8	2.4
HUAWEI TalkBand B2	2.5	–	2	3.7	2.1
Misfit Shine	4.5	77	3.5	4.2	3.2

For the preferences of users on the features of fitness trackers, the users look forward to expanded health and fitness features and are becoming more confident and approving of fitness trackers. As the prices continue to drop and the features continue to expand and improve, fitness trackers are becoming more attractive to consumers. The Ericsson Consumer Lab found that 40% of fitness tracker users indicated that they felt unsafe when not wearing fitness trackers and 43% of consumers believed that fitness trackers would replace smartphones in the future (Lab, 2016). This increasingly positive user feedback indicates that, despite the many issues associated with the use of the current fitness trackers, consumers are confident about the future of fitness trackers. Among the available features of fitness trackers, the top three preferences of users were daily activity tracking, heart health monitoring, and professional fitness tracking. Another consumer survey showed that the top three preferences of users were heart health monitoring, daily pedometer, and sleep monitoring. For the health management features, the top three preferences of users were heart rate monitoring, daily pedometer, and professional fitness tracking. Another consumer survey showed that the top three preferences of users were heart rate monitoring, Electrocardiography (ECG) monitoring, and blood oxygen saturation monitoring. Trial users generally preferred the practical features of fitness trackers, and general consumers were more interested in the advanced features, suggesting that the trial users had good experience with and approved of the existing features. Despite certain incomparable characteristics between the sample populations, this study included evaluation of seven devices with more than 30 experienced users for each device. Thus, the results were representative of the preferences of users for the features of these fitness trackers. It should be noted that all populations value health and fitness applications, which will be expanded in the future to include new features such as security, smart clothing, and non-verbal communication.

## Conclusion

Users generally had positive subjective intent regarding fitness trackers but were unsatisfied with their cost effectiveness. More users were willing to wear than to buy the devices. This is because wearable devices only provide these health data and do not provide the corresponding medical meanings or suggestions which only enables users to get data but does not know how to use these data to customize their fitness plans or even find out their own health risks. The wearable device at this stage only provides the role of recording data, which makes the user think that the cost performance of the wearable device is generally low. Take Xiaomi as an example; due to more health functions and thus higher cost performance, it has increased market share significantly from 34.3% in 2014 to 42% in 2015, Tencent ISUX. The users were more interested in daily activity tracking, heart health monitoring, and professional fitness tracking. In order to increase cost-effectiveness, wearable device related technologies should be combined with specialized health institutions such as hospitals and clinics to actively provide health services to users.

## Supplemental Information

10.7717/peerj.5350/supp-1 Supplemental Information 1 Survey data analysis. Sheet 1: Questionnaire results.
Sheet 2: The percentage of subjects who were willing to wear and buy each device (%).
Sheet 3: Different equipment evaluation system score.
Sheet 4: Variance analysis of scores for different systems of various devices.
Sheet 5: Respondents’ functional preferences.
Sheet 6: Subject’s functional preferences for health management.
Sheet 7: The price of the wearable devices.
Sheet 8: The coefficient in the comprehensive scoring model, Contribution rate, Weights of wearable devices.

10.7717/peerj.5350/supp-2 Supplemental Information 2 Questionnaire results summary.

10.7717/peerj.5350/supp-3 Supplemental Information 3 Smart wearable device comprehensive evaluation questionnaire.

## List of Abbreviations

EE: Energy Expenditure
LCD: Liquid Crystal Display
VR: Virtual Reality
AR: Augmented Reality
SUS: System Usability Scale
ECG: Electrocardiography

## Additional Information and Declarations

### Data Availability

The following information was supplied regarding data availability: The raw data are provided in the Supplemental Files.
