# Supplementary material for: Perceived user preferences and usability evaluation of mainstream wearable devices for health monitoring
Source: PeerJ. 2018 Jul 25;6:e5350. doi: 10.7717/peerj.5350 (PMC6064199; doi:10.7717/peerj.5350)
Supplement: Supplemental Information 3 [file peerj-06-5350-s003.docx]

# Smart Wearable Device Comprehensive Evaluation Questionnaire

# Ⅰ Your Information

1. **Sex**

Male

Female

1. **Age**

under 18

18~25

26~30

31~40

41~50

51~60

up to 60

1. **Education level**

| Junior high school and below |
| --- |
| Secondary and high school |
| College |
| Undergraduate |
| Master  PhD |

1. **Profession**

| IT Internet |
| --- |
| financial |
| medical health |
| education |
| manufacturing  FMCG  education  legal  Trade consumption  service industry  Advertising media  Agriculture, Forestry, Animal Husbandry and Fishery  other industry |

1. **Monthly income（¥）**

| <1000 |
| --- |
| 1000~2000 |
| 2001~4000 |
| 4001~7000 |

>7000

1. **Have you used any wearable device?**

Never

Yes, is___________

# Ⅱ Overall awareness of wearable devices

1. **Evaluation equipment**

Apple watch

Samsung Gear S

Fitbit Surge

Jawbone Up3

Jawbone Up2

Misfit(shine/flash)

Mi band

HUAWEI Talk Band B2

Misfit Shine

Leixin K.Band

1. **I will use this product often.**

Agree 1 2 3 4 5 Disagree

1. **I don't think this product needs to be so complicated.**

Agree 1 2 3 4 5 Disagree

1. **I think this product is easy to use.**

Agree 1 2 3 4 5 Disagree

1. **I feel that I need experienced people to help me to use this product.**

Agree 1 2 3 4 5 Disagree

1. **I think the various features of this product are well integrated.**

Agree 1 2 3 4 5 Disagree

1. **I think there are too many inconsistencies in this product.**

Agree 1 2 3 4 5 Disagree

1. **I think most people can quickly learn to use this product.**

Agree 1 2 3 4 5 Disagree

1. **I think this product is very troublesome to use.**

Agree 1 2 3 4 5 Disagree

1. **During use, I feel confident.**

Agree 1 2 3 4 5 Disagree

1. **In order to operate this product I need to learn a lot.**

Agree 1 2 3 4 5 Disagree

# III Product Design Rating

1. **Product design**

- Comfortable to wear

Very dissatisfied

Not satisfied

general

satisfaction

Very satisfied

- Industrial design level

Very dissatisfied

Not satisfied

general

satisfaction

Very satisfied

- Material affinity for skin

Very dissatisfied

Not satisfied

general

satisfaction

Very satisfied

- Key distribution rationality

Very dissatisfied

Not satisfied

general

satisfaction

Very satisfied

1. **Durable level**

- Degree of fever

Very dissatisfied

Not satisfied

general

satisfaction

Very satisfied

- Battery life

Very dissatisfied

Not satisfied

general

satisfaction

Very satisfied

- Work fineness

Very dissatisfied

Not satisfied

general

satisfaction

Very satisfied

- Material stability

Very dissatisfied

Not satisfied

general

satisfaction

Very satisfied

1. **Usability**

- Setting complexity

Very dissatisfied

Not satisfied

general

satisfaction

Very satisfied

- See statistics convenience

Very dissatisfied

Not satisfied

general

satisfaction

Very satisfied

- System response speed

Very dissatisfied

Not satisfied

general

satisfaction

Very satisfied

- Rationality of operation logic

Very dissatisfied

Not satisfied

general

satisfaction

Very satisfied

1. **Additional features**

- Practicality

Very dissatisfied

Not satisfied

general

satisfaction

Very satisfied

- Application scalability

Very dissatisfied

Not satisfied

general

satisfaction

Very satisfied

- Human-computer interaction intelligence

Very dissatisfied

Not satisfied

general

satisfaction

Very satisfied

- Supports connection device compatibility

Very dissatisfied

Not satisfied

general

satisfaction

Very satisfied

1. **reliability**

Sports data record

1 2 3 4 5

Energy consumption monitoring

1 2 3 4 5

Sleep node monitoring

1 2 3 4 5

Automatically identify the type of exercise

1 2 3 4 5

# IV Subjective feelings about the product

1. **Are you satisfied with the function of the device?**

Very dissatisfied 1 2 3 4 5 very satisfied

1. **Would you like to wear this device for a long time?**

willing

Unwilling, because_________

1. **Are you willing to buy this equipment?**

willing

Unwilling, because_________

1. **If you do not consider the ability to pay, how much do you think it is more appropriate to purchase this equipment?**

____________

1. **Device highlights**

The most favorite aspects_________________

Some of the least favorite aspects_____________________

Can further improve the device's recommendations___________________
